# Access to Water Source, Latrine Facilities and Other Risk Factors of Active Trachoma in Ankober, Ethiopia

**DOI:** 10.1371/journal.pone.0006702

**Published:** 2009-08-20

**Authors:** Ilya Golovaty, Larissa Jones, Bizu Gelaye, Melkie Tilahun, Habtamu Belete, Abera Kumie, Yemane Berhane, Michelle A. Williams

**Affiliations:** 1 Department of Epidemiology, Multidisciplinary International Research Training Program, University of Washington School of Public Health, Seattle, Washington, United States of America; 2 Addis Continental Institute of Public Health, Addis Ababa, Ethiopia; 3 Addis Ababa University, Addis Ababa, Ethiopia; University of California Los Angeles, United States of America

## Abstract

**Objective:**

This study aims to determine the prevalence and correlates of active trachoma in Ankober, Ethiopia.

**Methods:**

A cross-sectional community-based study was conducted during July 2007. A total of 507 children (ages 1–9 years), from 232 households were included in the study. All children were examined for trachoma by ophthalmic nurses using the WHO simplified clinical grading system. Interviews and observations were used to assess risk factors. Logistic regression procedures were used to determine associations between potential risk factors and signs of active trachoma.

**Results:**

Overall, the prevalence of active trachoma was found to be 53.9% (95%CI 49.6%–58.2%). Presence of fly-eye (fly contact with the eyelid margin during eye examination) (Odds Ratio (OR) = 4.03 95% CI 1.40–11.59), absence of facial cleanliness (OR = 7.59; 95%CI 4.60–12.52), an illiterate mother (OR = 5.88; 95%CI 2.10–15.95), lack of access to piped water (OR = 2.19; 95%CI 1.14–6.08), and lack of access to latrine facilities (OR = 4.36; 95%CI 1.49–12.74) were statistically significantly associated with increased risk of active trachoma.

**Conclusion:**

Active trachoma among children 1–9 years of age in Ankober is highly prevalent and significantly associated with a number of risk factors including access to water and latrine facilities. Trachoma prevention programs that include improved access to water and sanitation, active fly control, and hygiene education are recommended to lower the burden of trachoma in Ankober, Ethiopia.

## Introduction

Trachoma is the leading cause of infectious blindness in the world [1]. It is most prevalent in developing countries where poverty, insufficient access to clean water, sanitation facilities and health resources are continuing problems. The World Health Organization (WHO) estimates that trachoma is endemic in 56 countries, most lying within Africa and the Middle East, and causes 3.6% of all blindness [1]. It is currently reported that close to 1.3 million people are blind from trachoma while about 84 million people suffer from active trachoma. Given the enormity of the disease, WHO launched the Alliance for the Global Elimination of Trachoma (GET) by 2020 using the ‘SAFE’ strategy[2],[3]. The strategy has four components: Surgery, Antibiotics, Facial cleanliness and Environmental sanitation[2], [3].

Trachoma is caused by infection with the *Chlamydia trachomatis* bacterium, may be transmitted by fomites, direct contact, and the eye-seeking fly *Musca sorbens* which lays its eggs on exposed human feces [4], [5]. Trachoma clusters within family members with increased risk among caretakers [6]. Notably studies have shown that women and young children (especially 1–9 years) are at the greatest risk for contracting and transmitting trachoma [3], [7].

The National Blindness and Low Vision Survey, conducted in 2006, has estimated that trachoma is the second major cause of blindness and the third major cause of low vision in Ethiopia [8], [9]. It is estimated that nearly 10 million children presently show clinical signs of trachoma [8], [9]. Previous research indicates that risk factors among this population are most commonly: infrequent face washing practices, numerous eye-seeking flies, crowding, lack of latrines, livestock in the household, lack of chimney and lack of garbage disposal facilities [10], [11], [12]. Factors most consistently associated with trachoma have been inadequate access to water and sanitation [13], [14]. There has also been evidence that altitude may be a risk factor; however this finding is contradictory [7], [15].

Recent studies in the Tigray region [16] and Amhara region [10] have shown that northern Ethiopia is hyperendemic for trachoma. With a prevalence of 62.6%, the region carries a disproportionate amount of the national burden of trachoma. The region also has the highest prevalence of active trachoma among children aged 1–9 years [9], [17].

The high burden of trachoma in the Amhara region calls for a further region-specific data and comprehensive efforts to evaluate risk factors of trachoma in designing and expanding intervention programs. The aim of this study was to determine the prevalence of active trachoma in Ankober Woreda and to assess the extent to which it is associated with access to water source, latrine facilities, and other risk factors. To the best of our knowledge, this is the first study to assess the burden of trachoma in Ankober Woreda. This paper supplements the prevalence statistics of active trachoma among children in this region and identifies modifiable risk factors that could help for the improvement of programs aimed at trachoma prevention.

## Materials and Methods

**Figure 1:**
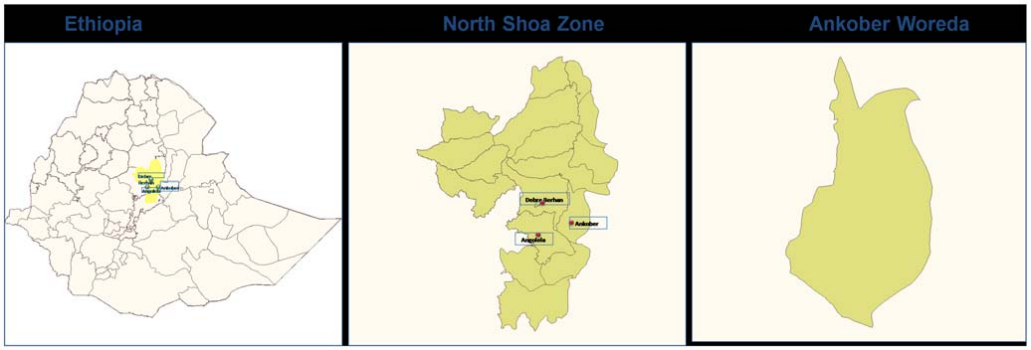
Map of Ankober Woreda, Ethiopia.

This cross-sectional epidemiologic study was conducted in Ankober Woreda, North Showa zone during the (rainy season) month of July 2007. Ankober is located 176 kms northeast of Addis Ababa, the capital city of Ethiopia (Figure 1: created using the WHO health mapper software). A group of eight kebeles (kebele is the lowest level of administrative unit in Ethiopia)[18] within 15-minutes walking distance from a water source were used to randomly select three kebeles: Gorebela, Aliyu Amba, and Aliyu Amba Zuria. An additional two kebeles, beyond 15 minutes of walking distance from the nearest water source, were chosen for the second group based on their accessibility by the survey team: Washa and Agere- Selam. In each selected kebele, households were selected using the proximity-sampling technique (a random direction from the center of the kebele was selected by spinning a pen. The households along that direction are then counted out to the boundary of the kebele, and one is then selected at random to be the first household surveyed. Subsequent households were selected as the next nearest until the desired sample size was reached). Household eligibility was determined by the presence of at least one eligible child of age 1–9 years in the household in accordance with the WHO trachoma prevalence indicators. All eligible children in selected households were included in the study. Children who were seriously sick at the time of the survey and did not fall in the correct age range were considered ineligible and excluded from the study. The final sample included 507 children (231 children from households within 15 minutes walking distance from water source; 276 children from households located over 15 minutes walking distance from water source) from a total of 232 households (118 households from kebeles within 15 minutes walking distance from water source; 114 households located at farther than 15 minutes of walk from water sources).

Eligible children were examined for the clinical signs of active trachoma using the WHO simplified clinical grading scheme [3], [19] by two trained and standardized ophthalmic nurses. In accordance with the WHO grading scheme, active trachoma was defined as the presence of either trachomatous inflammation-follicular (TF) or trachomatous inflammation-intense (TI). Each child was also examined for trachomatous trichiasis (TT) (defined as either inturned eyelashes rubbing on the eye or evidence of previously removed lashes) and trachomatous scarring. Those children who were found to have active trachoma were given three tubes of 1% topical tetracycline ointment (TTC) eye ointments and instructions on proper use were provided.

Individual and household risk factors were assessed using structured interviews with the heads of household. Individual and household risk factors were also assessed using observations. The head from each selected household was interviewed. In case of the household head absence, the second household head (husband or wife of the household head) were interviewed. Data were collected using a structured questionnaire, translated and printed in the Amharic language, consisting of information on socioeconomic, sanitary, environmental, and demographic risk factors. The survey included questions concerning: mother and father literacy (no, yes), access to latrine facilities (no, yes), waste disposal type (burned/bury, dumped in the farm, dumped in open space), clean face (no, yes), water source (piped, spring, or river/lake water), presence of fly-eye(fly contact with the eyelid margin during eye examination) (no, yes) and area of waste disposal (near house, away from house). Before the trachoma screening, a face examination was carried out by a team member, not involved in trachoma screening, to assess ocular (no, yes) and nasal (no, yes) discharge as well as the presence of flies. A clean face was defined as the absence of ocular and nasal secretions. Flies on the face was coded as ‘yes’ if a fly landed anywhere on the child's face within a 3 second window.

Data collection was conducted by two teams consisting of two ophthalmic nurses, two interviewers (male and female) and a local field guide who visited all the five kebeles. The two ophthalmic nurses were not involved in the interview data collection. The household data collectors obtained consent from the head of household or other adult member of the family. Interviews were begun by offering greetings, team member introductions, and explanations of the survey purpose.

Data were entered using EPI INFO (Version 3.3.2), a public access software made available from the US Centers for Disease Control and Prevention (CDC Atlanta, GA, USA) and the analysis was completed using SPSS (version 14.0, SPSS, Inc., Chicago, USA) and Stata (version 10.0, Stata Corp., College Station, TX, USA). Frequency distributions of study participants were explored and chi-square tests were used to determine differences between groups. Cross tabulations were used to describe the frequency distribution of characteristic among the sample population. Potential risk factors were categorized into socioeconomic (e.g., mother/father literacy), sanitary (e.g., facial cleanliness, presence of fly-eye), demographic (e.g., gender, age) and environmental factors (e.g., type of water source, latrine access) and analyzed accordingly. Logistic regression procedures were used to estimate multivariate adjusted odds ratios (OR) and 95% confidence intervals (95% CI) of active trachoma. Logistic regression was performed using the ROBUST command in STATA to adjust for our sampling scheme and clustering of cases within households. All reported p-values are two-tailed and statistical significance was set at 0.05.

### Ethical Consideration

Ethical approval for the study was granted by the Institutional Review Board (IRB) of Ethiopian Public Health Association (EPHA). Verbal consent was obtained from parents or appropriate guardians of eligible children before they were included in the study in accordance with the principles of the declaration of Helsinki. Written consent was not deemed appropriate, given the low literacy rate in Ankober Woreda and the research involved no more than minimal risk to the subjects. Study procedures including oral consenting process were approved by the IRB of EPHA. Documentation of verbal consent was initiated and dated by the interviewers on data collection forms as approved by the IRB. Before analysis, personal identifiers were removed from each data set. The Human Subjects Division of the University of Washington, USA granted approval to use the de-identified and anonymised data set for analysis.

## Results

Characteristics of the five villages (*kebeles)* included in this study population are provided in Table 1. The two villages further than 15 minutes to a water source did not have access to piped water and relied on spring water (60.3% and 0.7%) and/or river or lake water (39.7% and 99.3%). Similar patterns were observed among these villages' inaccessibility to a latrine (2.4% and 0%).

**Table 1:** Characteristics of study sample in Ankober, Ethiopia, July 2007.

	Walking Distance from Water Sources				
	Villages within 15 minutes			Villages beyond 15 minutes	
	Gorobela	Aliyu Amba	Aliyu Amba Zuria	Washa	Agere Selam
Number of Children (507)	72	80	79	126	150
**Water source**	N (%)	N (%)	N (%)	N (%)	N (%)
Piped water	72 (100.0)	77 (96.3)	24 (30.4)	0 (0.0)	0 (0.0)
Spring water	0 (0.0)	3 (3.8)	41 (51.9)	76 (60.3)	1 (0.7)
River or lake water	0 (0.0)	0 (0.0)	14 (17.7)	50 (39.7)	149 (99.3)
**Access to latrine**					
No	15 (20.8)	20 (25.0)	74 (93.7)	123 (97.6)	150 (100)
Yes	57 (79.2)	60 (75.0)	5 (6.3)	3 (2.4)	0 (0.0)
**Disposal of waste**					
Away from house	42 (58.3)	75 (93.8)	33 (41.8)	116 (92.1)	98 (65.3)
Near house	30 (41.7)	5 (6.3)	46 (58.2)	10 (7.9)	52 (34.7)
**Area of waste disposal**					
Burn/bury	35 (48.6)	18 (22.5)	0(0.0)	0(0.0)	7 (4.6)
In the farm	3 (4.2)	6 (7.5)	44 (55.7)	25 (19.8)	40 (26.7)
Dump in open space	34 (47.2)	56 (70.0)	35 (44.3)	101 (80.2)	103 (68.7)

The overall prevalence of active trachoma in the study population was 53.9% (95% CI 49.6%–58.2%). Age was statistically significantly associated with active trachoma (P<.001). Presence of fly-eye (P<.001), absence of facial cleanliness (P<.001), an illiterate mother (P<.001), an illiterate father (P<.001), lack of access to piped water (P<.001), and lack of access to latrine facilities (P<.001) were statistically significantly associated with increased prevalence of active trachoma (Table 2).

**Table 2:** Active trachoma in relation to sociodemographic, personal, household and village characteristics in Ankober, Ethiopia, July 2007.

Characteristics	Trachoma Follicles (TF)		Trachoma Intense (TI)		Active Trachoma	
	n (%)	*P-value	n (%)	*P-value	n (%)	*P-value
**Age(years)**						
1	12 (5.4)	0.012	10 (7.5)	0.008	17 (6.2)	<0.001
2	17 (7.6)		12 (9.0)		20 (7.3)	
3	15 (6.7)		5 (3.7)		18 (6.5)	
4	15 (6.7)		9 (6.7)		19 (6.9)	
5	57 (25.6)		33 (24.6)		69 (25.1)	
6	25 (11.2)		15 (11.2)		31 (11.3)	
7	27 (12.1)		17 (12.7)		34 (12.4)	
8	25 (11.2)		7 (5.2)		27 (9.8)	
9	30 (13.5)		26 (19.4)		40 (14.5)	
**Gender**						
Female	129 (57.8)	0.674	86 (64.2)	0.045	163 (59.3)	0.222
Male	94 (42.2)		48 (35.8)		112 (40.7)	
† **Presence of fly-eye**						
No	98 (43.9)	<0.001	59 (44.5)	<0.001	122 (44.5)	<0.001
Yes	125 (56.1)		74 (55.5)		152 (55.5)	
** **Facial cleanliness**						
No	176 (78.9)	<0.001	105 (78.4)	<0.001	211 (76.7)	<0.001
Yes	47 (21.1)		29 (21.6)		64 (23.3)	
**Mother Literate**						
No	213 (95.5)	<0.001	131 (97.8)	<0.001	264 (96.0)	<0.001
Yes	10 (4.5)		3 (2.2)		11 (4.0)	
**Father Literate**						
No	194 (87.0)	<0.001	122 (91)	<0.001	239 (86.9)	<0.001
Yes	29 (13.0)		12 (9)		36 (13.1)	
**Water source**						
Piped water	43 (19.3)	<0.001	21 (15.7)	<0.001	51 (18.5)	<0.001
Spring water	62 (27.8)		44 (32.8)		77 (28.0)	
River or lake water	118 (52.9)		69 (51.5)		147 (53.5)	
**Access to latrine**						
No	200 (89.7)	<0.001	126 (94)	<0.001	250 (90.9)	<0.001
Yes	23 (10.3)		8 (6)		25 (9.1)	

* P-value from Chi-Square test.

† Fly-eye is defined as contact with the eyelid margin or tissue internal to the lid margin during the time taken to prepare for examination and examine the child.

** A child's clean face was defined as the absence of ocular and nasal secretions on the face.

**Figure 2:**
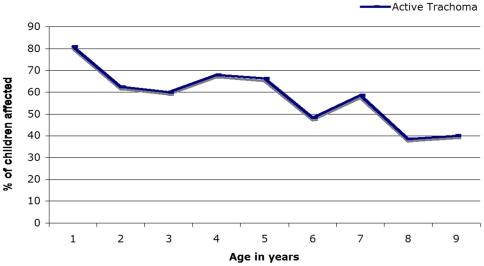
Prevalence of active trachoma by age in Ankober, Ethiopia, July 2007.

As shown in Table 3, logistic regression analysis showed that the risk of active trachoma decreased with increased age. Children under the age of three had nearly a two and half times greater risk of active trachoma than children over the age of seven (OR = 2.42; 95% CI 1.38–4.27). Figure 2 shows an overall decreasing trend of active trachoma with age among children 1–9 in the sample population; there is a peak of prevalence at 1 year and an increased in trachoma at year 4 and 7. Gender was not statistically significantly associated with risk of active trachoma. Factors related with child cleanliness were associated with active trachoma. Children with dirty faces were over 7 times more likely to have active trachoma than children with clean faces (OR = 7.59; 95% CI 4.60–12.52). Children with fly-eye were 4 times more likely to have active trachoma (OR = 4.03; 95% CI 1.40–11.59), as were children without access to a latrine (OR = 4.36; 95% CI 1.49–12.74). Children with illiterate mothers were almost 6 times more likely to have active trachoma (OR = 5.88; 95% CI 2.10–15.95) whereas children with illiterate fathers had no significant association with increased risk of active trachoma (OR = 1.61; 95% CI 0.53–4.96). Children who only had access to river or lake water had a greater than doubling in risk of active trachoma (OR = 2.19; 95% CI 1.14–6.08), as compared with those children who had access to piped water.

**Table 3:** Active trachoma in relation to socio-demographic, sanitary and environmental characteristics in Ankober, Ethiopia, July 2007.

Characteristics	Number of Active Trachoma Cases	Number	Active Trachoma
Overall	232	507	**Odds Ratio (95%CI)
***Demographic***			
**Age(years)**			
1–3	17	21	2.42 (1.38–4.27)
4–6	38	62	1.93 (1.39–2.67)
7–9	67	170	1.00 (Reference)
**Gender**			
Female	163	288	1.19 (0.79–1.79)
Male	112	219	1.00 (Reference)
***Socioeconomic***			
**Mother Literate**			
No	264	441	5.88 (2.10–15.95)
Yes	11	66	1.00 (Reference)
**Father Literate**			
No	239	402	1.61 (0.53–4.96)
Yes	36	105	1.00 (Reference)
***Sanitary***			
* **Facial cleanliness**			
No	211	260	7.59 (4.60–12.52)
Yes	64	247	1.00 (Reference)
† **Presence of fly-eye**			
No	122	323	1.00(Reference)
Yes	152	180	4.03 (1.40–11.59)
***Environmental***			
**Access to latrine**			
No	250	382	4.36 (1.49–12.74)
Yes	25	125	1.00 (Reference)
**Water source**			
Piped water	51	173	1.00(Reference)
Spring water	77	121	1.79 (0.51–6.37)
River or lake water	147	213	2.19 (1.14–6.08)

† Fly-eye is defined as contact with the eyelid margin or tissue internal to the lid margin during the time taken to prepare for examination and examine the child.

* A child's clean face was defined as the absence of ocular and nasal secretions on the face.

** Separate models for demographic, socioeconomic, sanitary, and environmental risk factors were used for analysis. Each variable is adjusted for age and other covariates in the model.

** Corrected to account for clustering within children living in the same Kebele

## Discussion

In this study of children 1–9 years in Ankober, we found that the overall prevalence of active trachoma was 53.9% (95% CI 49.6–58.2%). Active trachoma was independently associated with proxy indicators of low socioeconomic status, facial cleanliness, fly-eye, parental literacy and source of water.

The association between active trachoma and access to a latrine facility is consistent with previous studies [11], [20], [21]. Latrines are essential in reducing exposed human feces, the main breeding ground for the trachoma transmission vector *M. sorbens* [22]. Therefore, inaccessibility to latrine facilities and exposed human feces are risk factors for the presence of fly-eye. We found a very strong association between active trachoma and the presence of fly-eye similar to what was found by other investigators [23].

An increased walking distance to a water source was found to be an indication of decreased access to latrine facilities and to piped water which characterized the rural kebeles in this study. This rural/urban contrast in resources reinforce the findings of the Ethiopian national eye survey conducted in 2006, which found that the prevalence of active trachoma was four times higher in rural areas (42.5%) compared to urban areas (10.7%) [9]. A study conducted by Mesfin and colleagues [16] found an association between time to water source and decreased access to water. Therefore, a lack of water availability and sanitation services in rural Amhara might explain the hyperendemic burden of active trachoma in these areas. Baggaley *et al.* in Tanzania found a strong association (at the furthest distance, OR = 3.56, 95% CI 2.47–5.14) with trachoma prevalence and distance to water source [13]. Piped water, as a proxy-measure for water access, may give households a greater volume of available water for general use and thus lower their risk of active trachoma [14]. Therefore, the association between lack of piped water and increased active trachoma prevalence found in our study may suggest that greater access to water, in combination with other factors, may help to lower the risk of trachoma. However, a study completed in The Gambia found that regardless of the amount of water available for consumption, families with trachoma used less water than those without it after controlling for distance to water, family size, and other socioeconomic factors [24]. Households who allocate more water for hygiene practices show lower prevalence of trachoma [14]. Furthermore, it's been reported that increased access to water does not necessarily translate to improved hygiene practices [14]. It is possible that the *allocation* of water for hygiene practices in the household is the most predictive of trachoma prevalence [14], [25]. However, there is still uncertainty as to exactly how factors related to water access (time and distance to water source, per capita use, effort) interrelate and which factor is the most significant contributing factor to trachoma prevalence [13], [15], [16].

In this study, we found that children with unclean faces were 7 times more likely to have active trachoma. This result is consistent with studies that identify the presence of ocular and nasal discharge as risk factors for the presence of fly-eye and active trachoma [11], [16], [23], [26]. A study in The Gambia found that children with nasal and ocular discharge had twice as many flies in their eyes, putting them a greater risk for active trachoma [27]. It is suggested that the *C. trachomatis* bacterium is more easily transmitted in poor hygienic conditions through contact with nasal and ocular secretions [10], [28]. While the cross-sectional design of most studies make the causal relationship between ocular/nasal discharge and active trachoma impossible to verify [26], numerous studies have found that clean faces and frequent face washing practices are associated with reduced prevalence of active trachoma [26], [29]. This suggests that improvement in face washing behavior can significantly reduce a child's risk of acquiring or transmitting the trachoma.

Literacy of the mother was also a statistically significant risk factor for active trachoma among children. These results are in agreement with a study conducted by Mesfin *et al*. which showed a significantly (P<.05) higher prevalence of active trachoma among illiterates (OR = 1.38; 95% CI 1.13–1.69) [16]. Literacy of the mother may be especially important because she is responsible for the caretaking of the children. An educated mother may be more aware of the benefits of hygiene practices to the health of her children compared to an uneducated mother [6].

In this study, gender was not found to be significantly associated with active trachoma. These results are in general agreement with the national survey that found no gender differences in active trachoma [9] and other studies which found no gender differences [17], [26]. However, sex differences among children have been found in some studies [15], [30] and there is evidence that female children may be at greater risk for active trachoma because of their role in taking care of younger children in the family [30].

Increasing age was found to be a statistically significant protective factor for active trachoma. The results of our study showed a decreasing pattern of active trachoma prevalence as a child gets older. Similarly, previous research has also shown a decrease in active trachoma prevalence with increasing age [11], [13], [16], [23]. Bailey *et al.,* in their study suggested that young age is a risk factor for active trachoma because of the close contact children have with each other which aids in transmission [31]. Furthermore, they reported that acquisition of immunity as a child grows older plays a part in decreasing the duration of disease episodes. These findings indicate that concentrating on child-level risk factors are the most important points for intervention.

Interpretations of our findings are limited by several factors. The cross-sectional study design makes determining causality impossible. Walking time to water source may be an incomplete measure of water access, as it does not take into account accessibility of household water to the child [13]. Future studies should consider identifying more information on quality of the water source; piped water may not necessarily be the safest water source. Compromised water quality may indicate a decrease in water volume that can be allocated for hygiene purposes. Additionally, access to a latrine does not necessarily translate to latrine use [32]. Indications of latrine use and water allocation practices were not recorded which may have caused us to overlook certain relationships between active trachoma and determined risk factors. Since this study was conducted in July, during the rainy season, and the *M. sorbens* population has been shown to peak at the end of the rainy season, the actual prevalence may be higher than what is estimated in this study [33], [34]. Given the non-random sampling strategy; the prevalence results from the present study may not apply to other Woredas in the Amhara region.

In conclusion, trachoma among children 1–9 years of age in Ankober is highly prevalent and significantly associated with a number of risk factors including access to water source and latrine facilities. Access to sanitary water and latrines are essential factors of the ‘E’ component of the SAFE strategy. Effective prevention programs should integrate education about proper latrine use [16] and promote equal utilization of latrines among both genders and all age groups [9], [32] in order to preserve the protective nature of latrines. Campaigns focusing on introducing educational intervention programs in women's organizations, schools, and communities [33] could help bring behavioral changes among caretakers and children and lower the burden of trachoma in the North Showa zone.
